# Challenges in developing broad-spectrum viral attachment inhibitors targeting heparan sulfates and sialic acids

**DOI:** 10.1128/jvi.00139-26

**Published:** 2026-03-02

**Authors:** Valeria Cagno

**Affiliations:** 1 Institute of Microbiology, Universite de Lausanne and University Hospital of Lausanne 27213, Lausanne, Switzerland; New York University Department of Microbiology, New York, New York, USA

**Keywords:** broad spectrum, glycomimetic, receptor, sialic acid, heparan sulfates, antiviral, entry

## Abstract

Many distinct viruses exploit cell surface glycans, particularly heparan sulfates and sialic acids, as initial attachment factors to facilitate entry into host cells. Because these interactions are highly conserved across diverse viral families, they have long been viewed as attractive targets for the development of broad-spectrum antiviral strategies. Over the past decades, numerous approaches have attempted to block these early binding events, including genetic or enzymatic removal of glycans from the cell surface, masking of cell surface glycans, the use of engineered decoy receptors, and the development of multivalent inhibitors. Despite promising *in vitro* results, no antiviral therapy based on this mechanism has yet advanced to routine clinical use. Here, the biological roles of heparan sulfates and sialic acids in viral entry are examined, and the range of antiviral strategies designed to interfere with these interactions is discussed. The major challenges that have limited clinical translation are highlighted, including insufficient potency, potential off-target effects, the risk of resistance, and challenges related to routes of administration. Finally, recent technological advances that may help overcome these barriers and enable the development of clinically viable viral attachment inhibitors are proposed.

## THE VIRAL ATTACHMENT RECEPTORS

Attachment to the host cell is a critical first step in the viral life cycle, as it determines host specificity and enables the virus to initiate entry, infection, and subsequent replication within the target cell. Numerous viruses use the same attachment receptors to bind to the cell surface, thereby increasing the likelihood of engaging a more specific entry receptor, or they directly use the attachment receptor to induce conformational changes that initiate access to the cytoplasm. Often, these attachment receptors are composed of glycans, with the two major classes being heparan sulfates (HS) and sialic acids (SAs). HS are negatively charged, linear glycan chains attached to a protein core; the resulting complexes are called heparan sulfate proteoglycans (HSPGs) and are inserted into the cell membrane or present in the extracellular matrix (1, 2). HSPGs perform diverse and essential functions. Within the extracellular matrix, they support basal membrane organization and facilitate cell adhesion and motility. In secretory vesicles, they help maintain the proper activity of stored molecules. At the cell surface, HSPGs bind cytokines, chemokines, growth factors, and morphogens, protecting them from degradation and forming storage sites or gradients that are crucial for development. They also act as endocytic receptors, regulating lysosomal degradation, nutrient uptake, and receptor internalization. Additionally, HSPGs enable chemokine transport across endothelial cells and mediate intracellular signaling or stress responses through shedding (1, 2).

SAs represent a family of nine-carbon monosaccharides typically found at the terminal ends of carbohydrate chains linked to glycoproteins and glycolipids on eukaryotic cell membranes. This terminal positioning makes them highly accessible for protein-ligand and receptor-ligand interactions. SAs are therefore involved in multiple physiological processes, such as cell adhesion, signal recognition, regulation of complement activation, contribution to innate and adaptive immune responses through interaction with sialic acid-binding immunoglobulin-like lectins (Siglecs), and of the stability of proteins (3). Additionally, they have been shown to play fundamental roles in development (4).

The interactions of HS and SA with viral glycoproteins differ markedly in strength, specificity, and function. Interactions with HS typically rely on associations with positively charged residues on the viral glycoprotein. Due to the high mutation rates of viruses, increased dependency on HS has been reported to arise from single-point mutations (5, 6). These mutations occur naturally (6) but are also frequent while propagating viruses on cell lines due to the high abundance of HS, resulting in changes in viral receptor usage, as observed for lymphocytic choriomeningitis virus (LCMV) (7). HS dependency can also result from a compensatory pathway, when the traditional entry receptor is absent, as described for rhinovirus 89 in cells lacking the primary receptor ICAM-1 (8). Similarly, the interaction with HS has been reported to be sufficient for herpes simplex virus 1 (HSV-1) entry into primary corneal cells, where the commonly used receptors—nectin and HVEM (herpesvirus entry mediator, a member of the tumor necrosis factor proteins)—are not expressed (9).

For most viruses, interaction with HS represents only an initial step that facilitates binding to a specific entry receptor. For example, even after binding to HS, the main entry receptor required by Enterovirus 71 is SCARB-1, with this interaction occurring at the endosomal level (6). Similarly, in the case of SARS-CoV-2, binding to HS has been shown to promote the open conformation of the spike protein, thereby enabling its interaction with the entry receptor ACE2 (10) and to induce clustering of ACE2 promoting cell-to-cell spread (11).

In contrast, interactions with SA are often more specific and can be sufficient to trigger viral entry, although some viruses use them only as attachment receptors to increase their density on the cell surface and subsequently interact with a specific proteinaceous receptor, such as reoviruses (12) and Middle East respiratory syndrome coronavirus (MERS-CoV) (13). SA-binding viruses display linkage-specific recognition of SA. For instance, human influenza strains preferentially bind α2,6-linked SA, whereas avian strains bind α2,3-linked SA (14, 15). Viruses can also display specific preferences for linear or branched glycans with terminal SA (16) and generally possess highly specific binding pockets (15) rather than clusters of positively charged amino acids, as is the case with HS binding.

The distinction between viruses that bind HS and those that bind SA is not straightforward, as several viruses have been reported to bind both, for example, parainfluenza virus 3 (PIV3) (17) and adenovirus 37 (Ad37) (18), albeit with different outcomes. PIV3 depends on both HS and SA for entry, and removal of either from the cell surface results in a decrease of infectivity (17). In contrast, for Ad37, interaction with HS can be detrimental to entry, as it prevents interaction with SA (18).

Importantly, a virus’s binding preference for a specific glycan is crucial during the initial attachment phase but can present drawbacks during the egress phase, due to undesired reattachment of newly produced virions to the host cell. For this reason, SA-binding viruses also express proteins with a neuraminidase function—such as in influenza virus (IV) and parainfluenza virus (PIV)—to cleave SA and facilitate viral release (19, 20). In contrast, for HSV, it has been shown that the virus upregulates cellular heparinase to cleave HS and overcome this critical step (21).

## ANTIVIRAL STRATEGIES TARGETING VIRAL ATTACHMENT

**Fig 1:**
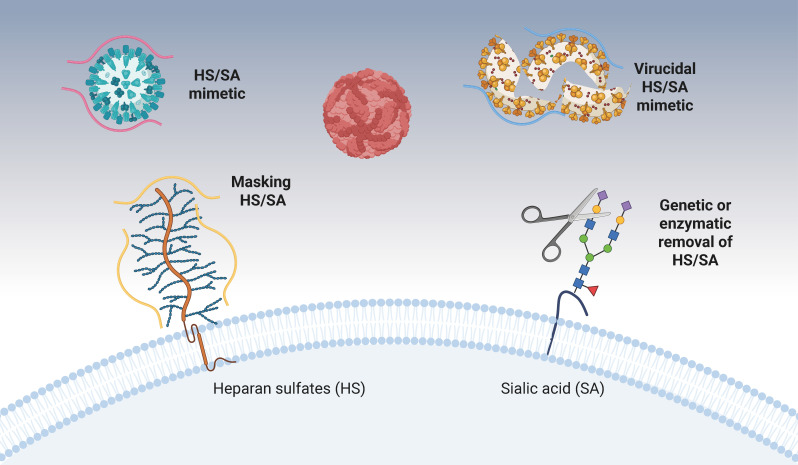
Antiviral strategies targeting viral attachment. Viral inhibition can be achieved by genetic or enzymatic removal of cellular receptors, by masking them, or by directly interacting with the virus through a virustatic or virucidal mechanism. HS, heparan sulfates; SA, sialic acid.

To block the interaction between the virus and the HS and/or SA on host cells, different strategies can be envisaged and are represented in Fig. 1, while the most advanced are summarized in Table 1.

**TABLE 1:** Attachment inhibitors with proven activity in human-derived tissue models, *in vivo* or in clinical trials^*c*^

Strategy	Molecule	Virus	Development phase	Administration^*d*^	Treatment regimen
SA removal	DAS-181 (22, 23)	PIV^*a*^^,*b*^	Phase III clinical trial	Dry powder for inhalation or aerosol	Therapeutic
		IV^*a*^	Phase II clinical trial	Dry powder for inhalation or aerosol	Therapeutic
Masking HS/SA	HEX17 (24–26)	IV^*a*^	Phase II clinical trial	Nasal spray	Preventive
		RSV^*b*^	Mouse	Intranasal	Preventive and therapeutic
		SARS-CoV-2^*b*^	Hamster	Intranasal	Preventive
	Vc4CBM (24)	PIV3^*a*^^,*b*^	Cotton rat	Intranasal	Preventive
	AGMA1 (27)	HSV-2^*b*^	Mouse	Intravaginal	Preventive
			Human-derived vaginal tissues	Apical	
	SB105A10 (28, 29)	RSV^*b*^, HMPV^*b*^	Human-derived airway tissues	Apical	Preventive
	G1 G2 peptides (30, 31)	HSV-1^*b*^	Mouse	Eye drop	Preventive
	Protamine sulfate (32)	HPV^*b*^	Mouse	Intravaginal	Preventive
HS/SA mimetic	Heparin (33)	SARS-CoV-2^*b*^	Phase II clinical trial	Aerosol	Therapeutic
	K5 derivatives (29, 34)	HMPV, RSV^*b*^	Human-derived airway tissues	Apical	Preventive
	Chitosan derivative (Cpd 17) (35)	SARS-CoV-2^*b*^	Mouse	Intranasal	Therapeutic
		RSV^*b*^	Mouse	Intranasal	Therapeutic
	PRO2000 (36)	HIV^*b*^	Failed in phase III clinical trial	Vaginal gel	Preventive
	Carrageenan (29, 37, 38)	HIV^*b*^	Failed in phase III clinical trial	Vaginal gel	Preventive
		HPV^*b*^	Phase II b clinical trial	Vaginal gel	Preventive
		HMPV^*b*^	Human-derived airway tissues	Apical	Preventive
	Cellulose sulfate (39)	HIV^*b*^	Failed in phase III clinical trial	Vaginal gel	Preventive
	S3 G4 6SL PAMAM (40)	IV	Mouse	Intranasal	Preventive
	PAA-YDS (41, 42)	IV	Mouse	Intranasal and aerosol	Preventive
Virucidal HS/SA mimetic	SPL7013 (43)	SARS-CoV-2^*b*^	Clinical trial	Nasal spray	Therapeutic
	Sulfonated nanoparticles (44)	RSV^*b*^	Mouse	Intranasal	Preventive
		HSV-2^*b*^	Human-derived vaginal tissues	Apical	Preventive
	Sulfonated CD (45)	HSV-2^*b*^	Mouse	Intravaginal	Preventive
			Human-derived vaginal tissues	Apical	Preventive and therapeutic
		RSV^*b*^	Human-derived airway tissues	Apical	Preventive and therapeutic
		ZIKV^*b*^	Human-derived vaginal tissues	Apical	Preventive
	Sulfated benzene	SARS-CoV-2^*b*^	Hamster	Intranasal and aerosol	Therapeutic
	Sialylated CD-6′SLN (46)	IV^*a*^	Mouse	Intranasal	Preventive and therapeutic
			Human-derived airway tissues	Apical	
	Sialylated CD-SA (47)	IV^*a*^	Mouse	Intranasal	Therapeutic
			Human-derived airway tissues	Apical	
	Sialylated and sulfonated CD-SLNT-SO_3_^−^ (17)	RSV^*b*^	Mouse	Intranasal	Preventive and therapeutic
			Human-derived airway tissues	Apical	Preventive
		IV^*a*^	Zebrafish	Injection in the swim bladder	Preventive
			Human-derived airway tissues	Apical	
		PIV3^*a*^^,*b*^	Human-derived airway tissues	Apical	Preventive
		SARS-CoV^*b*^	Human-derived airway tissues	Apical	Preventive
	Star polymers (48)	RSV^*b*^	Mouse	Intranasal	Therapeutic
	ZP12 polymer (49)	Chikungunya virus^*b*^	Mouse	Intraperitoneal	Therapeutic

^*a*^ SA dependency.

^*b*^ HS dependency.

^*c*^ HIV, human immunodeficiency virus; HMPV, human metapneumovirus; HPV, human papillomavirus; HSV, herpes simplex virus; IV, influenza virus; PIV, parainfluenza virus; RSV, respiratory syncytial virus.

^*d*^ Intranasal, direct instillation; aerosol, nebulization for lung administration; nasal spray, droplets for nasal administration.

### Removal of HS and SA from the cell surface

HS and SA can be removed from the cell surface by genetic manipulation of the host cell. Indeed, multiple CRISPR/Cas9 screenings have demonstrated the dependency of viruses on the HS and SA biosynthetic pathways (50). For instance, the inactivation of the SA transporter *SLC35A1* drastically reduces the infection of enterovirus D68 (51) and of several strains of IV (52). Similarly, the inactivation of various genes involved in the HS biosynthetic pathway has been shown to reduce infection by different viruses, including SARS-CoV-2 and human coronavirus OC43 (53) as well as distinct orthoflaviviruses (54).

This approach is valuable for identifying the attachment receptors utilized by viruses. Indeed, cells lacking heparan sulfate expression, such as CHO psg-D677 and psg-A745 cells, are largely used to study the receptor dependence of multiple viral strains (9, 55, 56). Likewise, to investigate whether a virus is dependent on HS, the sulfation of these glycans can be reduced by sodium chlorate treatment, which inhibits the synthesis of the sulfate donor (57). Instead, sialylation of glycans can be reduced through metabolic inhibition of sialyltransferases, for example with 3-Fax-peracetyl Neu5Ac; this strategy has been evaluated in an antiviral context for IV and coronavirus OC43 (58).

As an alternative, viral attachment receptors can be enzymatically removed from the cell surface. An advanced approach to prevent and treat infections caused by SA-dependent viruses is DAS-181 (Fludase) (Table 1). This recombinant fusion protein consists of a sialidase from *Actinomyces viscosus* linked to an anchoring domain derived from human amphiregulin, which enables binding to glycosaminoglycans (59). DAS-181 has demonstrated *in vitro* efficacy against PIV and IV and has been evaluated in clinical trials. It decreased IV yield, measured by RT-qPCR in a phase II study involving 177 participants, although without significantly improving clinical symptoms (22). In a different phase II randomized clinical trial involving immunocompromised patients with PIV infection, DAS-181 did not meet the primary endpoint of survival at day 45; however, it showed benefit in a subset of severely immunocompromised patients improving lung function and facilitating weaning from oxygen supplementation (23). A phase III clinical trial is currently ongoing to assess its use in this specific patient population; however, to date, the results are not available. Broader application of DAS-181 appears unlikely due to treatment-related adverse effects, including immune stimulation and systemic absorption leading to circulating sialidase activity and elevated alkaline phosphatase levels observed in a phase I clinical study in healthy volunteers (59). These aspects limit both prolonged and repeated use in the same patient (59), thereby restricting its possible use.

Based on current knowledge, a comparable strategy involving recombinant heparinase has not been explored, likely owing to the enzyme’s reported pro-carcinogenic activity (60) and the potential risk of enhanced viral release from infected cells (21), while there is interest in inhibiting the host heparinase activity to limit viral detachment from the cell (61). Nevertheless, heparinase is widely used to investigate viral dependence on HS (6, 62).

### Masking HS and SA on the cell surface

Continuing with approaches that directly target the host, another strategy is to bind and mask HS or SA on the cell surface to prevent viral attachment. This is the rationale behind the design and development of HEX17 (Neumifil) (Table 1). This molecule is composed of hexavalent carbohydrate-binding domains derived from the *Streptococcus pneumoniae* NanA sialidase fused to a trimerization domain derived from a *Pseudomonas aeruginosa* pseudaminidase, resulting in an oligomer with six sialic acid-binding sites per molecule. The macromolecule was then further optimized to reduce its predicted immunogenicity *in vivo*.

HEX17 can bind and mask SA residues on the cell surface, as well as directly interact with viral glycoproteins. The preventive activity of HEX17 against various respiratory viruses has been demonstrated both *in vitro* and *in vivo*, including IV, SARS-CoV-2, human rhinovirus, and respiratory syncytial virus (RSV) (24). While the mechanism is clear for SA-dependent viruses, the inhibition of non-SA-dependent viruses may result from steric masking of neighboring sites of sialic acid on the cell surface or from direct interactions of HEX17 with viral glycoproteins. In particular, an interaction of HEX17 with ACE2 and DPP4, the main receptors of SARS-CoV-2 and MERS-CoV, as well as with the glycoproteins of SARS-CoV-2 and MERS-CoV was shown (24, 25).

This compound was evaluated in a phase II randomized, placebo-controlled clinical trial in 99 adults who received HEX17 either once before being challenged with IV or three times (2.8 mg/administration) or a placebo control. The treatment resulted in a significant reduction in symptomatic infection for both arms, with 40% symptomatic influenza in the placebo-treated group versus 20.3% in the pooled-treated groups (26). Additionally, a significant reduction in the mean viral load curves from day 1 to day 8 was observed, by both RT-qPCR and viral culture (26). However, no clear difference was observed between the two arms with single and repeated administration, and further analysis should evaluate the efficacy in a clinical trial with natural exposure rather than in challenged individuals. Further studies should investigate the potential therapeutic use of HEX17 and assess its efficacy against additional viruses, especially the ones not dependent on sialic acid, to better understand the translatability of animal findings to humans.

Other molecules were also reported to mask HS on the cell surface. For instance, the cationic polymer AGMA1 was shown to inhibit HSV-1, HSV-2 (27), and human papillomavirus (HPV) (63) by binding to HS and preventing viral attachment. The polymer was shown to be effective in human-derived vaginal tissue models and *in vivo* (27) (Table 1), while derivatives were shown to be active against human immunodeficiency virus (HIV) in vitro (64). A dendrimer, SB105A10, was also shown to inhibit multiple HS-dependent viruses *in vitro* by interacting with HS, with an activity conserved as well on human-derived respiratory tissues against RSV (28) (Table 1). However, these molecules were not further studied in animal models or in clinical trials.

A different approach exploited a phage display library to identify peptides masking HS and 3-OS-HS on the cell surface. Two peptides, called G1 and G2, proved to be active against different strains of HSV-1 and HSV-2 and prevented viral binding. Additionally, the same peptides were also tested *in vivo* in a model of corneal infection with HSV-1 where a prophylactic topical administration proved active (30). Subsequently, the authors also evaluated in an *ex vivo* model of pig corneal tissues the combination of the G2 peptide and acyclovir, but the combination of the two drugs did not show a superior performance if compared with the single agents (31).

Finally, protamine sulfate (PS), an FDA-approved drug able to interact with heparin and reverse its anticoagulant effects, was also evaluated for its antiviral activity. When tested against HPV, PS proved to be active *in vitro* preventing viral attachment and productive entry, and in a mouse model when administered intravaginally one hour before infection (32).

### Mimicking HS and SA

So far, the approaches targeting the host have been described. These strategies carry a higher risk of side effects but offer the advantage of a higher barrier to resistance. However, when considering the development of a broad-spectrum antiviral intended for use in a large population and for acute infections, the reduced side effects associated with targeting the virus itself might be prioritized.

As already demonstrated by HEX17, a common feature to achieve good half-inhibitory concentrations by targeting viral attachment is multivalency, that is, the multiple presentation of the targeting moiety on the compounds to increase avidity for the attachment receptors or to the viral receptor binding domain. The most famous example of a multivalent HS mimicking molecule is heparin, which proved to be effective *in vitro* against multiple HS-dependent viruses (1). Heparin has been tested up to clinical trials; for instance, during COVID-19, a meta-trial showed positive effects of inhaled unfractionated heparin on intubation and death (33) (Table 1). However, the main limitation of heparin, when administered systemically, is linked to its possible side effects, especially the anticoagulant activity (65), and its interaction with many other positively charged peptides and molecules in the human body.

From heparin, a long series of sulfated and sulfonated molecules has been developed, including heparin derivatives devoid of anticoagulant activity, dendrimers exposing sulfonates and sulfates, carrageenans, and polymers with activity mostly at the attachment and entry phase of distinct viruses (1).

Among these, K5 polysaccharide derivatives show structures similar to heparin but with selective sulfation patterns. Their activity in preventing viral attachment has been demonstrated against a wide range of HS-dependent viruses (66); in particular, they were shown to be active against HMPV (29) and RSV (34) in a preventive setting in human-derived respiratory airway models.

Recently, chitosan derivatives were shown to inhibit SARS-CoV-2 and RSV in cells and *in vivo* also when administered post-infection (35). However, the molecules that advanced to clinical trials almost invariably failed to retain the antiviral activity demonstrated *in vitro*. PRO2000, a sulfonated polymer, was evaluated in a phase III clinical trial as a topical microbicide for HIV prevention; although it was shown to be safe, it failed to show any efficacy (36). Similarly, Carraguard, a carrageenan-based compound, was shown to be ineffective in a separate clinical trial for HIV (37), but showed 37% protection in a trial for the prevention of HPV, while it did not demonstrate activity in clearance of already acquired infections (38). Meanwhile, cellulose sulfate not only failed to prove effective in an HIV prevention clinical trial, but also appeared to increase the infection rate, despite having previously shown effectiveness in animal models (39).

Similarly to multivalent inhibitors of HS-dependent viruses, multivalent SA-mimicking molecules have also been evaluated. The most extensively studied target is IV, for which, in addition to multivalency, ligand density and spacing have been investigated in greater detail (67). Since IV hemagglutinin is a trimer, several studies have shown that an optimal multivalency of three ligands, with appropriate spacing, enables effective interaction with the individual monomers (40, 68). Some of these inhibitors showed efficacy both *in vitro* and *in vivo*, such as multivalent 6′-sialyllactose-polyamidoamine (6SL-PAMAM) conjugates with a spacing of 3 nm between each sialic-acid-terminating sugar, which demonstrated activity in preventive settings in mice when administered intranasally (40). Synthetic polyacrylamide-based sialylglycopolymer PAA-YDS also showed activity *in vitro* and *in vivo* against IV following intranasal and aerosol administration; however, concerns were raised that degradation of the polymer core could potentially lead to toxic byproducts, and probably for this reason, these molecules were not further pursued (41, 42)

### Mimicking HS and SA with a virucidal activity

Several years ago, structural features that can significantly enhance the potency of attachment inhibitors by conferring a virucidal activity were identified. Achieving permanent viral inactivation requires that the compounds incorporate an active ligand mimicking the attachment receptor, linked via a hydrophobic moiety to a multivalent scaffold. This class of materials demonstrated the ability to damage the viral envelope, as evidenced by electron microscopy analysis (44, 47), DNA exposure assays (45), and envelope integrity assays by lipophilic dye unquenching (17, 47). This discovery led to the development of sulfonated nanoparticles (44), sulfonated cyclodextrins (45), sulfated benzenes (69), sulfonated polymers (70), and sialylated cyclodextrins (46, 47) and benzenes (69). The sulfonated and sulfated derivatives exhibit broad-spectrum antiviral activity, extending even beyond HS-dependent viruses (71), whereas the sialylated molecules primarily target IV strains, albeit with greater potency than the sulfonated, resulting in robust *in vivo* efficacy against IV maintained even when administered 24 or 48 h post-infection (46, 47). Finally, a single macromolecule mimicking both HS and SA was synthesized, further expanding the antiviral spectrum to encompass all major respiratory viruses. This molecule exhibited activity in human-derived respiratory epithelia against PIV3, RSV, SARS-CoV-2, human and avian strains of IV, and showed *in vivo* efficacy against RSV and IV (17).

Virucidal materials were also described previously; however, the characteristics needed for virucidal activity were not rationally investigated. For instance, a sulfated dendrimer, SPL7013, has been shown to cause a permanent inactivation of both HIV and SARS-CoV-2, demonstrated by the loss of infectivity after ultracentrifugation to separate the compound and the virus (72, 73). The dendrimer has been tested in several forms as an antiviral. Although it showed *in vitro* activity against various viruses, including HIV (73) and HSV (74), its *in vivo* efficacy has not been demonstrated, and in a phase I clinical trial, a gel containing the dendrimer (VivaGel) was associated with mild inflammation and irritation (75). The same dendrimer is also the active ingredient in a nasal spray, which has shown *in vitro* activity and *in vivo* activity against SARS-CoV-2 (76). Furthermore, it was evaluated in a double-blind, placebo-controlled trial, in which it reduced SARS-CoV-2 viral load in the upper respiratory tract of individuals over 45 years of age who did not require hospitalization (43). Nevertheless, additional testing is needed to evaluate its efficacy in individuals with more severe symptoms and to assess the broad-spectrum efficacy and its potential use in a preventive setting.

Recent work, still in preprint form at the time of writing, shows virucidal sulfonated polymers promising *in vivo* activity against RSV when administered topically (48) and, of particular interest, a zwitterionic form has demonstrated broad-spectrum *in vitro* activity and efficacy against Chikungunya virus when administered intraperitoneally, resulting in reduced viral load and alleviated joint swelling (49).

## CHALLENGES OF ATTACHMENT INHIBITORS

While the attachment receptors used by viruses have been known for a long time, targeting or mimicking them as an antiviral strategy is not yet a commercial option. Understanding the limitations of these approaches can help guide the future development of attachment inhibitors toward a more direct path to clinical use.

### Cell surface modification

Removing HS and SA from the cell surface has been a powerful tool for studying the biology of virus; however, the application as an antiviral strategy presents some limitations. Genetic approaches require an intracellular effect which is potentially more toxic than antiviral strategies acting extracellularly. However, ongoing developments in *in vivo* gene editing and gene silencing could potentially improve their feasibility. Currently, the best option available to remove SA from the cell surface is DAS-181. Although it is not a small molecule, it works extracellularly and has reached phase III clinical trials and has demonstrated efficacy in phase II trials in specific subpopulations, such as immunocompromised patients with severe respiratory infections caused by PIV (23). However, as previously discussed, its use in larger populations remains unlikely.

### Microbicide trial failures

Focusing on small molecules, the failure of several clinical trials investigating HIV prevention with microbicides whose active ingredients were sulfated compounds (36, 37, 39), as well as the limited efficacy observed for HPV (38), raises several considerations. The lack of efficacy may reflect challenges associated with vaginal administration, where physiological conditions and the presence of highly charged semen during intercourse may increase viral infectivity and hinder the action of microbicides (77). Additionally, for clinical microbicides, adherence in trials is difficult to measure (78), and formulations must maintain persistent antiviral activity, while subclinical mucosal irritation from the microbicide or its application can significantly affect infection outcomes, complicating the scenario (79). This explanation is indirectly supported by the fact that various approaches have shown *in vivo* efficacy in preventive and therapeutic settings for respiratory viruses (26, 33), whereas in clinical trials and *in vivo* models, topical microbicides demonstrated limited to no efficacy in preventive settings and no effect on patients or models who were already infected—for example, in clearing HPV (Table 1).

### Virucidal activity

The failure of microbicide clinical trials could be ascribed as well to the virustatic nature of the interaction between attachment inhibitors and viruses. The vaginal microbicides may have failed because their active components interacted with viruses only reversibly. Upon dilution in bodily fluids, the concentration of the antiviral agent therefore could have become ineffective. Even if there is no direct proof of this causal link in clinical trials, virucidal materials, which induce irreversible inactivation of viruses upon contact, demonstrated that this behavior results in more potent inhibition of several viruses, not only in cultured cells but also in human-derived tissues and in animal models (17, 44–46, 48, 49). The increased potency associated with virucidal activity also results in sustained inhibitory effects even when the molecules are administered in a therapeutic setting, that is, after infection has already occurred (17, 46–48). Therefore, future work on attachment inhibitors should prioritize the development of molecules with virucidal activity.

### Administration

Another major limitation to consider is the widespread distribution of HS and SA throughout the body and their essential physiological functions. Therefore, antivirals must achieve high concentrations at the site of infection to minimize side effects and maintain efficacy, as off-target interactions with host molecules can reduce their antiviral activity. This limitation also explains why, so far, the most advanced viral attachment inhibitors are administered topically; for instance, DAS-181 is formulated for delivery to the pulmonary surface via dry powder inhalation or nebulization (23), while PRO2000 and Carraguard were tested in clinical trials as vaginal microbicides (36–38). Also, the most promising results with unfractionated heparin were obtained through topical administration, and their effects are certainly not solely attributable to the antiviral activity (33). Indeed, when administered systemically—or even topically—HS-mimicking molecules can exert effects entirely independent of their antiviral activity, such as anticoagulant or anti-inflammatory effects. These effects may also contribute to improved disease outcomes, but they are unrelated to the mechanism of action discussed in this review (80, 81). Specific approaches must be explored to make this class of inhibitors effective when administered orally. This will be particularly important in the context of a pandemic, allowing the treatment to be easily administered even outside of a hospital setting (82). So far, only a preprint showed the systemic efficacy of a virucidal polymer against Chikungunya virus (49), but additional evaluation of the mechanism of action should elucidate whether the activity *in vivo* is linked solely to a direct effect on the virus.

An effective attachment inhibitor administrable systemically will open the possibility of targeting additional viruses, such as viruses with a viremic phase, for which so far only *in vitro* evidence is available, such as orthoflaviviruses or Ebola virus (1).

### Resistance

When developing an antiviral molecule, it is important to consider the risk for resistance, which appears to be different for HS and SA mimetics. In particular, HS-mimicking molecules have a high barrier for resistance, possibly linked to the presence of multiple positive amino acids on the cell surface and subsequent multiple mutations needed to diminish the HS dependency. However, for some sulfated molecules, resistance has been reported, with the loss of positive charges in the glycoprotein (83). In contrast, due to the higher specificity of sialic acid—viral glycoprotein interactions, the barrier to resistance is lower, and, for instance, point mutations close to or within the sialic acid binding site can result in diminished potency of the sialic acid mimicking molecules, as shown for IV hemagglutinin (17, 84). To avoid or delay the emergence of resistance, combined therapy could be envisaged, as previously shown by combining IFN-lambda and a SA-mimicking molecule (47). However, not every combination can result in this effect as demonstrated by the emergence of resistance with the combination of SA-mimicking molecules and baloxavir marboxil or oseltamivir (84).

### Broad spectrum

A distinct aspect to consider is the breadth of activity. Ideally, an antiviral should be active against the widest possible range of viruses infecting the same anatomical site, enabling treatment of multiple pathogens through a single route of administration. Since many respiratory viruses use either HS or SA as attachment receptors, by targeting or mimicking both simultaneously, an antiviral compound with a broad spectrum of inhibition can be developed. Such an approach could be crucial for preventing and treating seasonal respiratory infections as well as newly emerging respiratory viruses. Recent work shows that it is indeed possible to combine the two types of ligands on the same scaffold while maintaining virucidal properties and preserving activity against multiple relevant respiratory pathogens—in cell culture, in respiratory tissues, and *in vivo* (17). Future work should focus on evaluating alternative scaffolds and optimizing the combination and ratio of ligands to further enhance both broad-spectrum and virucidal activity.

## CONCLUSION

In conclusion, despite the challenges and limitations inherent to targeting viral attachment, the development of attachment inhibitors as broad-spectrum antivirals remains a critical area of investigation. The advent of artificial intelligence in drug discovery and the screening of large libraries of compounds could accelerate the identification and design of new antivirals capable of overcoming the limitations described (85). Advancing this class of therapeutics holds the potential to provide a powerful and versatile tool in the fight against both established and newly emerging viral threats, offering a new strategy to enhance global preparedness for future outbreaks.
